# Optimality Principles in the Regulation of Metabolic Networks

**DOI:** 10.3390/metabo2030529

**Published:** 2012-08-29

**Authors:** Jan Berkhout, Frank J. Bruggeman, Bas Teusink

**Affiliations:** 1 Systems Bioinformatics, AIMMS, VU University, 1081 HV, Amsterdam, The Netherlands; 2 Kluyver Centre for Genomics of Industrial Fermentation/NCSB, 2600 GA, Delft, The Netherlands; 3 Regulatory Networks Group, Netherlands Institute of Systems Biology, Amsterdam, The Netherlands; 4 Life Sciences, Centre for Mathematics and Computer Science (CWI), 1098 XG Amsterdam, The Netherlands

**Keywords:** metabolic regulatory networks, optimal regulation, systems biology, design principles

## Abstract

One of the challenging tasks in systems biology is to understand how molecular networks give rise to emergent functionality and whether universal design principles apply to molecular networks. To achieve this, the biophysical, evolutionary and physiological constraints that act on those networks need to be identified in addition to the characterisation of the molecular components and interactions. Then, the cellular “task” of the network—its function—should be identified. A network contributes to organismal fitness through its function. The premise is that the same functions are often implemented in different organisms by the same type of network; hence, the concept of design principles. In biology, due to the strong forces of selective pressure and natural selection, network functions can often be understood as the outcome of fitness optimisation. The hypothesis of fitness optimisation to understand the design of a network has proven to be a powerful strategy. Here, we outline the use of several optimisation principles applied to biological networks, with an emphasis on metabolic regulatory networks. We discuss the different objective functions and constraints that are considered and the kind of understanding that they provide.

## 1. Introduction

The availability of genome-wide datasets is increasing rapidly. Surprisingly, more data does not, per se, lead to better understanding. In other words, a mechanistic understanding of biological networks that are used to generate these datasets is lacking behind. One reason for being so is the emergent behaviour that arises from the interactions within these networks. Consequently, the systemic consequences of molecular perturbations cannot be predicted, nor does it lay bare the molecular mechanisms leading to cellular behaviour. Systems biology develops methodology to overcome these limitations and supplements molecular cell biology. Systems biology aims to understand the (dys-) function of living organisms by studying the dynamics of molecular networks; effectively by answering how molecular interactions give rise to cellular behaviour [1,2,3]. 

To achieve understanding of the function of a molecular network and how this results from molecular interactions requires the identification of the molecular make-up as an obvious first (and important) step. This often involves whole-genome sequencing, additional biochemical knowledge (“legacy data”) and experiments. Such approaches have led to the determination of the topology of metabolic networks for a great number of organisms [4,5]. Yet, how those molecular networks are rewired during adaptive responses, *i.e.*, how they are regulated and why, is less well understood [6]. This problem is so challenging because it requires the integration of signalling, gene, and metabolic networks. Metabolic networks alone are already composed of thousands of interactions between enzymes and metabolites. 

**Figure 1:**
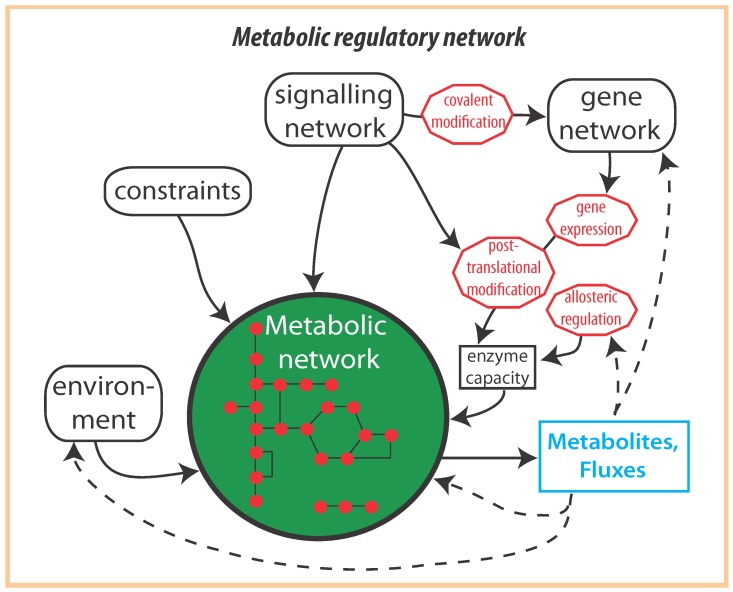
Overview of regulatory interactions involved in metabolic regulatory networks. The function of metabolic networks are governed by constraints. The regulation of a metabolic network involves a tight interplay between different cellular networks such as signalling and gene networks and by interactions with its environment. The enzyme capacity is the net result of the amount of enzyme expressed and its activity as dictated by post-translational modification and allosteric regulation. Metabolite pools and fluxes are considered as the outputs of metabolic reaction networks and can be involved in various regulatory feedback loops to other networks within the metabolic reaction networks as indicated by the dashed arrows.

The activity of metabolic networks results in a complex manner from internal constraints (such as enzyme kinetics, physico-chemical parameters, thermodynamics) and the interplay between environment and the impact of the associated signalling and gene network. Together these networks form, what we will call, the metabolic regulatory network (MRN, Figure 1). This complex network displays multiple overlapping regulatory interactions and feedback regulation. The recognition of this complexity has inspired many studies to integrate datasets from different cellular regulatory levels [7,8,9,10,11,12]. These and other studies often address **how** activities or components are affected by environments. Yet, a very different question is **why** this particular behaviour is there in the first place. Is it possible to understand the *design principles* of MRNs? We think we can, and we think that such understanding will help in finding the generic biological structures underlying the piles of data that are being gathered with todays technologies. 

**Figure 2:**
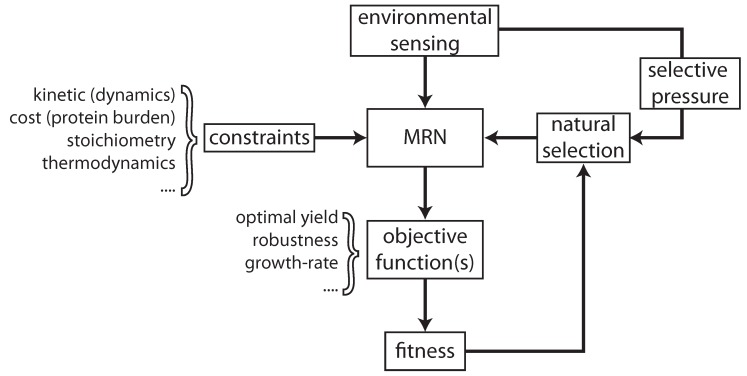
Schematic overview of the interactions involved in the process of evolutionary optimization of metabolic regulatory networks. Constraints limit the functionality of a metabolic reaction network (MRN), which for a given environmental condition can be analysed with respect to (a) certain objective function(s), giving rise to some fitness. Depending on selective pressures (which in turn are also dependent on the environment), natural selection acts on the fitness of a metabolic reaction network.

Figuring out the design principles of a MRN compares to a reverse engineering strategy. In order to design a certain device, an engineer needs a system specification saying what the system should do—what its function is—and all the relevant constraints should be considered, e.g. the production costs, life-time of the device, *etc.* When one studies a biological design, the reverse approach is required. The function—arising from the “specs”—and the relevant constraints need to be identified. When a similar function occurs across organisms or networks and when those are achieved by similar networks, a design principle is found. In other words, a design principle can be seen as the mechanism for network functions that have proven to be successful in evolution. Such principles likely have emerged as the outcome of similar selective pressures. Thus, in biology, it makes sense to identify design principles as the result of an optimisation process of (a determinant of) fitness. The outcome of an optimisation of a given fitness measure and/or a given set of constraints is then taken as a hypothesis that can either be falsified or verified [13]. This process will be discussed in this review (Figure 2). 

We thus start from the fact that adaptations are an inherent property of all living organisms, which is to be explained by natural selection. However, that does not mean that the adaptations are always perfect, nor that fitness is always optimal, only that fitness changes in a direction of increase. Thus, we try to gain insight by studying specific examples of adaptation, in the light of natural selection and historical interactions and constraints (see also [13,14] and a classical paper criticising optimisation approaches by Gould and Lewontin [15]). 

**Figure 3:**
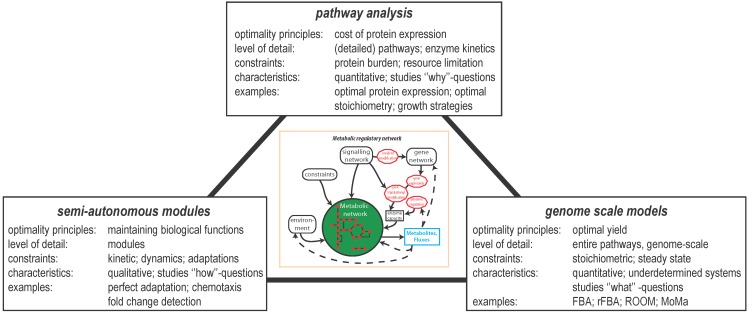
Different approaches to study metabolic regulatory networks classified according to the level of detail. Depending on the level of detail of analysis, different objective functions can be addressed. **Pathway analysis** refers to the analysis of (detailed) reactions that might be embedded into a pathway. With this type of analysis **why** questions can be addressed. **Semi-autonomous modules** function independently of the rest of the network, and have a discrete function. Concepts from control and information theory have been applied to understand **how** functionality emerges from the molecular components of these modules. **Genome scale models** uses the information content from the entire genome to figure out **what** flux distribution may lead to an optimal behaviour.

An example of one such optimality hypothesis is the economy of protein expression. The typical large number of enzymes in metabolic networks results in a significant burden on total cellular resources [16,17,18,19]. Cellular fitness can only increase if the right enzymes are expressed at the right time. Thus, the regulation, or the lack thereof, of metabolic networks can have large beneficial or adverse effects on cellular fitness, as shown experimentally [18,20,21]. Alternatively, biological systems have been exploited to achieve other control objectives. Examples in bacteria include: optimisation of growth rate [22,23,24]; optimal swimming pattern [25,26]; adequate timing of transcription of amino acid metabolic enzymes [27]. The above examples have in common that they all use an optimality principle to understand systems behaviour. Some approaches, both theoretical and experimental, aim to yield fundamental insights into the design principles of MRNs. Other approaches, such as flux balance analysis (FBA) simply assume such design principles exist and explore optimality to predict behaviour at a genome-scale. We discuss the relative advantages and limitations of these approaches, with a focus on the type of optimality hypothesis that is relevant (Figure 3). Finally, some open questions regarding MRNs are provided. 

## 2. Optimal Control of Metabolic Reaction Networks for Semi-Autonomous Modules

Biological functions can rarely be attributed to single molecules; instead it is the result of many interacting molecules within cells. A convenient way of studying biological function is therefore by the analysis of biological modules [28,29]. As proposed by Hartwell *et al.* [30] “Modules are composed of many types of molecules. They have discrete functions that arise from interactions among their components,... , but these functions cannot easily be predicted by studying the properties of the isolated components.” Inspired by the similarities between biological modules and man-made objects, analyses from control and information theory has been applied to biological modules, yielding insight in the general principles that govern the function of biological systems, including MRNs [12,26,29]. One can think of a module as a sub-network inside the cell that can carry out some task nearly irrespectively of the state of the remainder of the molecular network inside the cell [31]. Examples of such systems are stress-response systems, (e.g., the heat-shock system and osmotic shock response), iron scavenging systems, or two-component signalling systems in bacteria. These systems function semi-autonomously inside cells. Even though modules occur within molecular networks, not all functions derive from them. For instance, current data does not suggest that metabolism can be perceived as a mosaic of *weakly coupled* modules engaged in mass exchange: one responsible for alanine synthesis, another for ATP generation, or for DNA synthesis. It is much more likely that metabolism operates as a unit with strong interdependencies between subnetworks that carry out different functions. This should also have consequences for its control and regulation by signalling and gene networks: *i.e.*, about the design principles of MRNs. 

### 2.1. Maintaining Biological Function in Dynamic Environments

In their natural habitat nearly all organisms encounter environmental dynamics that require adaptation in order to improve fitness. These observations can have different interpretations with respect to the aim and consequences of the networks involved. Here, we will argue that although adaptations are indispensable, they are not the objective. Rather, it is maintaining biological functions (in varying environments), which is in the end achieved through adaptations [14]. 

Robustness is a fundamental property of biological systems [32,33,34,35,36]; it allows a biological system to perform its functions despite external or internal perturbations. This necessitates robust systems to behave in a flexible way upon disturbances without affecting the performance/function of the perturbed network. All changes should compensate for reductions in function upon perturbation. In other words, robustness is a mechanism that allows changes in the structure and components of the network, while specific functions of the network are maintained. While robustness is a key feature of biological systems, they should at the same time be evolvable; e.g., large changes in fitness should be possible as a result of only a few mutations. This is an intriguing aspect of biological systems; robust to specific perturbations while evolvability is maintained. In addition, a system which is robust to all types of perturbation is thus likely to lead to an evolutionary conservatism that inhibits the discovery of new adaptive solutions [37]. 

**Figure 4:**
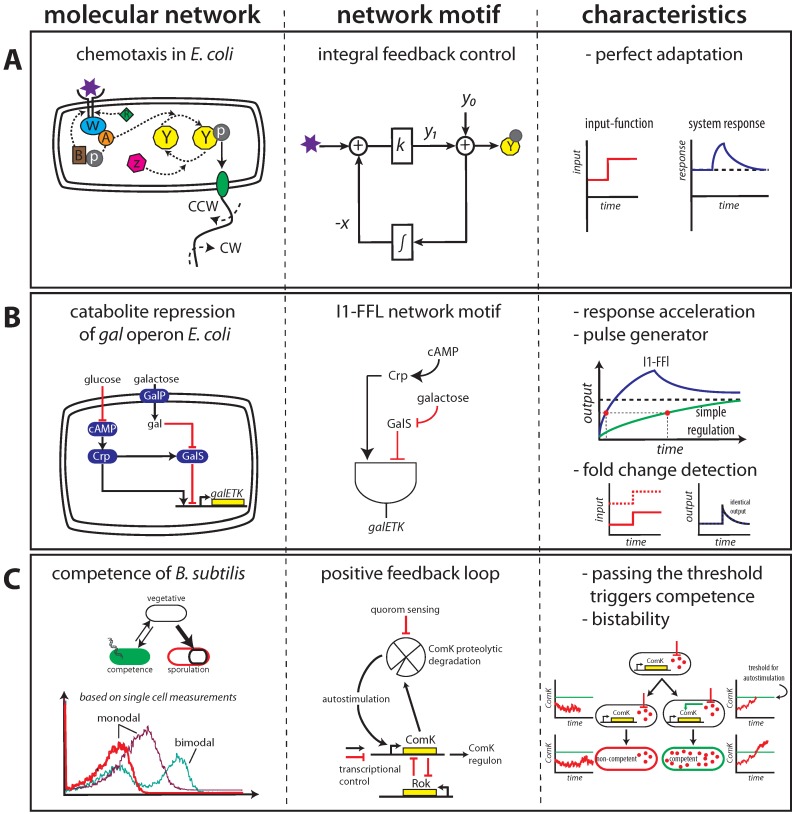
Illustration of network motifs to study molecular networks. In the left column the molecular interactions underlying the networks are shown, the middle column shows the network motif and corresponding characteristics are plotted in the right column. The examples shown here, are discussed in detail in the main text. **A** Chemotaxis signalling network in *E. coli*. (Figures adapted from [26,46]). **B** Catabolite repression of the *gal* operon in *E. coli*. (Figures adapted from [31]). **C** Competence in *Bacillus subtilllis*. (Figures adapted from [47,48]).

Cells often achieve robustness through feedback circuitry or the activity of a signalling network, which sense environmental changes and induce compensatory or adaptive responses. Many characteristics of signalling modules have been studied by analysing their input-output relationship. A feature displayed by several signalling modules is perfect adaptation. Arguably the best studied module for perfect adaptation is bacterial chemotaxis in *Escherichia coli*. The signalling network in bacteria responsible for chemotaxis has served as a classical example for understanding how functions at the network level emerge from interactions between constituent molecular components. In this network, as shown in Figure 4A, changes in attractant or repellent concentrations (purple star) are sensed by a protein complex consisting of transmembrane receptors, adaptor protein CheW (blue), and a histidine kinase CheA (orange). Autophosphorylation activity of CheA is inhibited by attractant binding and enhanced by repellent binding to receptors. The phosphoryl group is transferred from CheA to the response regulator CheY (yellow). The level of phosphorylated CheY (Y-P) influences the direction of the flagellar motors. Adaptation is mediated by the action of two enzymes: CheR (green) and CheB (brown), which adds or removes methyl groups on each receptor monomer. Feedback is provided by CheB phosphorylation through CheA that increases CheB activity. Chemotaxis allows bacterial cells to swim in the direction of favoured chemical attractants (such as food) or away from repellents (such as toxic compounds) by changing the frequency of tumbling [38,39] (for reviews on chemotaxis see [40,41] and additional references therein). Interestingly, the mechanism for chemotaxis is based on a robustness feature called perfect adaptation (Figure 4A) [25,26,35,36]. The steady-state behaviour of the system is independent of the concentration of the attractant or repellent, which is achieved by resetting the output value to the pre-stimulus level. Barkai and Leibler showed, using computer simulations, that perfect adaptation is not dependent on the fine-tuning of parameters in the network; instead, it was shown that perfect adaptation was an intrinsic property of the connectivity of the signalling network [35]. Experimental evidence for this robustness property in chemotaxis was provided by Alon *et al.* [36]. In an elegant experimental set-up these authors showed that perfect adaptation was not affected when the levels of chemotactic proteins were changed over a wide range. This inspired other studies to apply concepts from control and information theory resulting in the observation that an integral control system is key for robust perfect adaptation [26,42,43]. This becomes evident when the molecular network is represented as a block diagram (Figure 4A), with the chemoattractant as input and receptor activity (Y-P) as output. −*x* represents the methylation state of the receptors. The difference between output (*y*_1_) and a reference value (*y*_0_) represents the error. Integral control arises through the feedback loop in which the integrated error is fed back into the system, leading to perfect adaptation. A recent computational search of all possible three-node networks which are able to perform perfect adaptation confirmed this observation [44]. 

Perfect adaptation was also experimentally demonstrated in the regulation of osmotic shock in yeast [45]. By means of single-cell analysis of the dynamic response of the hyper osmotic shock network, these authors demonstrated that nuclear enrichment of a MAP kinase (Hog1) displays perfect adaptation. The requirement of an integral feedback system to observe perfect adaptation was exploited to reveal the importance of Hog1 kinase activity. 

In contrast to perfect adaptation, another observed adaptive response is fold change detection (FCD) [43,49,50]. In FCD the dynamics of the output is only dependent on the fold-change in the level of the input signal, and thus not on the absolute levels of the signal. A system that displays FCD will produce identical outputs (not only the magnitude of the response peak but also the entire profile over time depends only on the change ratio of the stimuli) in response to, for instance, a change in input signal from 2 to 4 compared to 10 to 20 (Figure 4B). Modules that display FCD should thus display some sort of memory in order to compare the change in input signal to the previous state. Recently, it was shown in a theoretical approach that FCD can be generated by a so-called incoherent feedforward loop (I1-FFL) [49]. In a I1-FFL, an activator, X, activates a repressor Y, that in turn control target gene Z, which is also directly activated by X. Other examples of network motifs that can display FCD are a nonlinear feedback loop and a linear integral feedback system [51]. 

What are the advantages of FCD? It is postulated that having a FCD (i) ensures an adequate response towards an activator that is known to naturally vary up to many folds and (ii) the ability to maintain sensitive despite noise in the input signal [49] (see also section 2.2). The latter becomes clear when considering two inputs: a low signal and a high signal. If noise is in some manner proportional to the level of signal, a high level of noise is unavoidable for the high signal, which might cause fundamental problems for absolute detection mechanisms. In other words, FCD allows organisms to respond optimally to a gradient and this response is invariant of multiplying the gradient with a constant [51]. 

Another characteristic of a l1-FFL motif is its ability to generate a pulse and accelerate the response. This behaviour is the result of the two opposing inputs (X activates Z and Y; Y represses Z) that are integrated by Z. Upon an increase in X, Z is rapidly increased, but after some time, Y builds up and starts to decrease Z again. As a result, initially Z rises rapidly, and then its concentration drops again, leading to pulse-like dynamics. An example of this motif can be found in the *gal* operon in *E. coli*: external galactose is transported into the cell by a permease (GalP). Internal galactose (gal) binds to gal isorepressor (GalS), which negatively regulates the *gal* operon. External glucose inhibits production of cAMP which, when bound to protein Crp, acts as an activator of the *gal* operon. I1-FFL speeds up a response (indicated by the red dots) and generates a pulse upon stimulation compared to simple regulation (Figure 4B). The acceleration of the response, up to 3-fold compared to a simple regulation system, arises when GalS does not completely inhibit *galETK*. Disruption of the l1-FFL motif, by mutations or artificial conditions, abolished these dynamics [52]. 

### 2.2. Managing and Profiting from Inevitable Molecular “Noise”

The molecules that make up biological networks are continuously synthesised and degraded, where the latter process results from intrinsic degradation and/or via dilution due to cell growth. Synthesis and degradation rates should be balanced to maintain a molecular species at a constant level over time. Due to the inevitable asynchrony in the individual synthesis and degradation events, fluctuations in molecule numbers are such that transient deviations from the steady state level occur. Those fluctuations occur in single cells and remain invisible when populations of cells are studied. When single cells were studied it became clear that molecular species, which occur in small numbers, such as transcription factors, can display large fluctuations—“noise”; the coefficient of variation (defined as the ratio of the standard deviation to the mean) ranges in 25%–40%. Even though such fluctuations are transient, they do cause alterations in network function because they induce variable protein expression levels. In this manner, fluctuations in molecular species can lead to phenotypic differences between genetically identical cells living in a homogeneous environment [53,54]. In depth discussions can be found in reviews about experimental approaches to noise in gene expression [55,56] or about the mechanisms generating population-level variability [57,58,59]. Here, we will focus on how cells cope with molecular noise, as sometimes they aim to minimise it, while in other cases they profit from it. Each of these cases requires particular network topological features and resulting dynamic behaviours. A few design principles have been discovered that indicate quite universal strategies for how cells cope and exploit noise. 

In many cases, molecular regulatory networks are inevitably distorted by noise—although it remains to be shown how much of a reduction in fitness it actually causes. One situation where noise can cause a reduction in cellular performance is when it occurs in signalling networks. Such systems respond to environmental changes and show changes in their output, e.g., a transcription factor, upon a change in external signal level. But, if the components of the signalling network spontaneously fluctuate in their level and their activation state, these systems can show changes in their output even at a constant signal level: this is when they make an error. This effectively reduces the capacity of cells to distinguish alternative environmental states. The number of distinguishable states can be related to the characteristic size of the change in the output level due to changes in the signal level divided by the size of the output change induced by spontaneous fluctuations at a fixed signal level. This measure thus compares the level of desired signal to the level of background noise and is called the signal-to-noise ratio; a central concept in information theory [60]. Recently, Cheong *et al.* [61] determined the number of environmental states that can be distinguished by the NFκB signalling network in mammalian states and found a surprisingly low number of only two states. Estimates by Tkacik and Bialek [60] show similar numbers for other systems, indicating that signalling networks are severely reduced in function by noise and that cells function despite this low performance. 

Cells can also profit from noise, particularly when they are confronted with unpredictable environments where sudden stress or food shortages can occur. One well-studied example is the sporulation and competence response of *Bacillus subtilis* upon sudden stress conditions [47]. Competence is a physiological state, distinct from sporulation and vegetative growth, that enables cells to bind and internalize transforming DNA. Based on single cell measurements, cells have either a single (low or high) unimodal or bimodal distribution of some indicator for competence (distributions shown as an example only, Figure 4C). Regulation of the activity of ComK, a transcription factor in the competence network, is controlled through proteolytic degradation, quorum sensing and transcriptional control. ComK binds to its own promoter and is required for its own expression. The competence regulatory network therefore contains a positive feedback loop. Due to the positive feedback loop ComK proteins (red circles) auto-stimulates itself (green arrow) after passing a threshold (green line) leading to competence. When the ComK concentration does not cross the threshold ComK levels remain low and cells stay in a non-competent state as indicated on the left side. Since the start, duration, and size of the stress is unclear and causes cell death, cells should prepare themselves and make sure that some of them survive—they all have the same DNA so altruistic behaviour can actually occur. In growing populations, a certain fraction of cells expresses the key regulator of competence ComK at high levels and acquire competence for DNA uptake while the majority of cells remain in the low ComK expression state. In this manner, a fraction of the population is prepared for the stress while others continue to produce offspring as long as the conditions allow for this and in this process make some more dormant cells. The exact probability for a cell to become dormant or proliferative is not fixed and is actually under cellular control. Whether a given cell becomes proliferative or dormant is a stochastic event achieved by molecular noise. This does not only occur in *B. subtilis* but also in other cells. The design principle here is that a bistable network is used that can switch between states due to spontaneous fluctuations in the levels of its molecular components (e.g., ComK expression level, Figure 4C), *i.e.*, stochastic phenotype switching. 

## 3. Global Pathway Analysis

### 3.1. Optimal Gene Expression in Un-branched Metabolic Pathways

The group of Heinrich pioneered the application of optimality principles to biological systems [62]. They studied the control structure of metabolic systems at states of optimal activity [63,64], the optimal timing of metabolic gene expression [65], optimal gene expression patterns were found under the assumption that expression patterns serve as regulators of cell functions [66], and tested whether metabolic network design and stoichiometry could be the outcome of an evolutionary optimisation process [65,67]. The optimal timing has been illustrated experimentally, whilst the other theories have inspired new ways of thinking about biological systems but await experimental proof—provided such experiments indeed can be done. 

In the aforementioned experimental study, Zaslaver *et al.* measured the promoter activity of amino-acid biosynthetic genes in *E. coli* over time, using GFP and Lux libraries [27]. Inspired by earlier work from Heinrich [65], these authors used a mathematical analysis to unravel the underlying objective of the observed timing of gene expression (a.k.a. just-in-time transcription). Based on their analysis, it was concluded that just-in-time transcription is beneficial when selective pressures act on rapidly reaching a new steady state with minimal enzyme production costs [27]. 

### 3.2. Playing the Optimality Game

Optimisation of organismal fitness is not always as straightforward as discussed so far. This has to do with the fact that optimal biological traits or designs should also be evolutionary stable. Excretion of extracellular proteins is such an intriguing trait. Let us take the excretion of extracellular protease by the lactic acid bacterium, *Lactococcus lactis*, as an example. This protease is involved in the degradation of milk proteins to form free utilisable peptides, as is needed to support growth, in conditions where they are not (or not enough) supplied directly into the medium. One might predict that, under such conditions, the trait of excreting the protease will be selected for; after all, excretion of the protease lead to more free peptides that are needed for cellular growth. However, since the proteases are excreted extracellularly, the peptides produced do not merely benefit the cell secreting the protease, but in part also diffuse away from it, becoming accessible to neighbouring cells. Imagine the scenario where one cell does not excrete the protease. This “cheater” cell does not have the burden of production and excretion, but is still able to take up free peptides. On the long term, this cheater can thus, on average, generate more offspring leading to an increase in the protease-negative trait. Ultimately, the trait can completely disappear in a population, as was shown experimentally [68]. Similar results were obtained in yeast that excretes invertase extracellularly. The invertase is involved in the breakdown of sucrose into glucose and fructose. The latter two are able to diffuse away from the cells that excreted the invertase, and therefore accessible to other cells [69]. These two examples are a very counterintuitive outcome of the effect of selection on the physiology of a species, even under constant conditions. A detailed theoretical analysis of this cooperative and cheating behaviour was reviewed recently [70]. In conclusion, when fitness of a phenotype is dependent on its frequency relative to other phenotypes in a given population, game theory approaches are useful tools because they take evolutionary stability of traits into account. 

### 3.3. Growth Rate Optimisation Shapes Growth Strategies

Cellular behaviour is governed by interacting networks. How is it possible to explain a global cellular property, such as the growth rate, if one studies only a sub-part of the network? This question inspired Molenaar *et al.* to propose a self-replicating model of a cell that is composed of a minimal set of required modules, such as a module for the ribosomes, metabolic pathways, substrate transporters and lipid biosynthesis [71]. The essence of this self-replicating model is: for a given environment, total cellular resources are optimally allocated by the ribosomes (including allocation to synthesis of new ribosomes), such that the growth rate is maximised. Using this approach, it was shown how the experimentally frequently observed shift between metabolically or energy efficient and inefficient metabolism can be explained as the result of optimising the cellular economy for growth rate. Other optimisation approaches, including FBA (see section 4.1) and game theory (see section 3.2), have failed to explain such shifts in metabolic strategies because they will always select one of the strategies irrespective of the external substrate level. 

### 3.4. Optimal Protein Expression Levels Maximise Growth Rate

Experiments in which enzyme levels were titrated using inducible promoters provide indirect but compelling evidence that microorganisms optimally tune enzyme levels [72,73,74]. Interestingly, these experiments showed that the growth rate of *E. coli* is maximal at the wild type level of ATPase expression, indicating that in *E. coli*—for the enzymes considered—protein levels are fine-tuned to result in the highest growth rate. Similar results have been described for glycolytic enzymes in *L. lactis* [75,76] as well as the activity of the *las* operon [77]. In addition, laboratory evolutionary experiments [78], by means of serial propagation [79], also confirmed that protein expression levels optimise cellular fitness. However, the resources a microorganism has at its disposal are limited. This implies that expressing and maintaining enzymes is costly. Indeed, it has been observed that expressing unneeded proteins has a negative effect on the growth rate [17,18,19]. Depending on the environmental conditions, protein expression can also be beneficial. Therefore, in order to optimise fitness, a precise balance between these two processes is required. 

Above examples show that mutations affecting the amount of protein expressed are an adaptive mechanism to deal with changing environments and thus a target for selective pressure to act on. However, these observations do not provide an explanation in terms of the underlying design principle. This was provided by Dekel and Alon, who proposed a quantitative model in terms of the underlying costs and benefit of protein expression [20]. Using the *lac* operon in *E. coli*, these authors were able to experimentally measure the cost and benefit of protein expression of *β*-galactosidase (LacZ), which hydrolyses lactose into glucose. While increasing the expression level of this enzyme will increase the cost, the benefit of its activity will increase with increasing lactose concentrations. Cost and benefit were measured by the effect of different protein expression levels on the growth rate at different lactose concentrations. The optimal expression level—that is, where the difference between benefit and cost is maximal—was measured for different lactose concentrations. Interestingly, a serial propagation evolution experiment, at different lactose concentrations, revealed that cells adapt their protein expression level to the predicted optimal level (within a few hundred generations) [20]. This indicates that, under the right environmental conditions, the selection pressure on a single protein can be very strong. 

Additionally, Eames and Kortemme recently presented an approach to perturb the production, function and folding efficiency in redesigned *E. coli* strains, and showed that the lac permease (LacY) activity is a major physiological source of expression costs in the *lac* operon [21]. The cost scales linearly with LacY activity (and not with LacZ) and outweighs cost-effects of protein production and misfolding in the *lac* operon. In light of the regulatory network of the operon, these results signify mechanisms that minimise the physiological costs of LacY activity, such as the direct inactivation of LacY function in the presence of glucose and other carbon sources, known as inducer exclusion. 

The concept of a cost-benefit optimisation has also been applied to explain gene regulation functions (the relation between input signals and the gene expression levels) of the *lac* operon [80]. Starting with a measured gene regulation function, and assuming that this function has evolved to optimally suit the natural environment of *E. coli*, the authors were able to explain the shape of the gene regulation function in terms of underlying regulatory mechanisms. For example, the steep shape of the regulation function at intermediate lactose levels is suggested to optimally minimise the effects of noise in an environment with a bimodal distribution of lactose concentrations. 

The definitions for cost and benefit as initially coined by Dekel and Alon [20] were explicitly given in terms of the *lac* operon kinetics for *E. coli*. In order to make such an analysis applicable to metabolic pathways, we recently presented generalised definitions for cost and benefit (Berkhout *et al.*, submitted). We propose quantitative and intuitive concepts for cost and benefit of enzymes in a metabolic pathway, and calculate these from kinetic and biochemical data, under the assumption that fitness increases with pathway flux. To test our predictions, a laboratory evolution experiment with *Saccharomyces cerevisiae*, propagated on galactose, under growth rate selection, was performed. Interestingly, the experimental data agree with the model predictions by showing that a high increase of growth rate is attained by increasing phosphoglucomutase levels, as observed previously as well [81,82]. The above examples show that a cost-benefit analysis can shed light on biological principles such as protein expression and gene regulation and provide a framework that carries strong predictive power. 

### 3.5. Feasibility Analysis

The regulation of metabolic networks is the net result of the interplay between modulation of enzyme levels [83] (sometimes called hierarchical regulation [7,84]), and metabolic regulation, e.g., via allosteric interactions. Quantitative analyses that relate changes in metabolic fluxes to changes in transcript or protein levels have revealed a remarkable lack of understanding of the regulation of metabolic networks. Combining the **why** and **how** questions might be a step towards obtaining a better understanding of the regulation of MRNs. Recently we have proposed such a method: feasibility analysis [85]. 

Feasibility analysis starts with a kinetic model of metabolism, of which the parameters are sampled to create solution spaces (which are bounded by constraints) and a (sub-) set of these samples is selected according to an objective function or multiple objective functions. Thus, feasibility analysis allows for (a visual) discrimination between different modes of metabolic regulation, and evaluates conditions where multiple objective functions have to be traded-off by cells. Using experimental data from long-term carbon limited chemostat cultivation of yeast cells [86], with feasibility analysis, we were able to understand the observed adaptations. We concluded that yeast cells, after long term adaptation, employ a mixed strategy between (at least) two opposing strategies: decreasing enzymes expression levels as a way to decrease enzyme overcapacity and investing in expression levels involved in taking up the limiting growth substrate. This trade-off renders the cells specialised in a low-carbon flux state to compete for the available glucose and get rid of the enzyme overcapacity. 

Based on ^13^C-flux experiments in different bacteria, it was recently shown that metabolism indeed operates close to the optimal surface defined by multiple objective functions: maximum ATP yield, maximum biomass yield and minimal sum of absolute fluxes [87]. Using flux data from evolved *E. coli* on alternating carbon sources, it was proposed that the location in the flux state space (the feasible space constrained by the three objective functions) can be understood from an underlying trade-off between (near-) optimality under a given condition and minimal adjustment to alternative conditions. 

An advantage of feasibility analysis is that the analysis is based on a kinetic model, therefore a more extensive set of objective functions can be implemented. An example is the objective functions that involve dynamics, which cannot be studied within FBA. Simultaneously, it limits feasibility analysis, due to the number of kinetic models available. Although a kinetic model of the entire metabolic network of an organism will be extremely useful to characterise fully the mechanics of each enzymatic reaction, and to combine such knowledge to ultimately predict system-behaviour, we are far from that. Besides some first attempts towards this goal [88,89], the number of complete kinetics models is sparse. Furthermore, kinetic enzyme assays are often done under *in vitro* conditions which do not always resemble *in vivo* environments [90,91,92]. For a depository of available and curated models of biological systems the reader is referred to online databases such as JWS [93] and Biomodels [94]. Note that the former also allows for the user to run these models online. 

## 4. Genome Scale Models

The arrival of whole-genome sequencing techniques has provided a comprehensive description of the genetic content (the “parts-list”) that makes up an organism. The reconstruction of all (metabolic) reactions within an organism requires the identification of all enzymes encoded by the genome and the chemical transitions that they participate in. A complete description of the entire genome was long thought to be *the* requirement to understand biological function (and ultimately to cure diseases). It turned out, however, that due to elaborate regulatory networks these promises could only be partially achieved. Nevertheless, these studies have facilitated the acceleration of the development of other experimental techniques (for instance ChIP-and RNAseq) as well as the development of an active field of research that develops genome-scale *in silico* models. These models are used to explore all flux distributions within the network which results in optimisation of an objective function. 

### 4.1. Flux Balance Analysis

Flux balance analysis (FBA), inspired by the premise that during evolution natural selection prompts biological systems towards optimality, assumes that cells perform optimally with respect to a given objective function. The optimisation of such an objective function is used to find a (sub-) set of optimal states from the large solution space that is defined by the constraints. The optimisation of the production of biomass or ATP are examples of commonly used objective functions. 

Does the optimality assumption within FBA help in understanding biological systems? Yes and no. Let us take the optimisation of specific growth rate (that is equivalent to maximisation of the rate of production of biomass per unit biomass [95]) as an example. There is considerable evidence that, especially under laboratory evolution experiments, the growth rate is representative of cellular fitness. So, how does FBA optimise the growth rate? FBA relates (measured) input and output rates via the equation: 


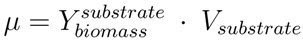
(1)

where µ is the specific growth rate (unit h^−^^1^), 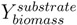 is the biomass yield with respect to the substrate (unit gram dry weight per mmol substrate, gDW mmol^−^^1^) and V_substrate_ is the specific uptake rate of the growth substrate (unit mmol h^−^^1^ gDW^−^^1^). With the uptake rate fixed as an environment-dependent capacity constraint, in order to find the highest µ, FBA just finds the flux distribution with the highest yield. Thus, it should be realised that the maximisation of growth rate is essentially done by finding a flux distribution that maximises 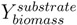 (see also [96]). Hence, FBA can rather accurately predict flux distributions for cases where high-yield strategies are favourable [97]; however predictions using FBA fail when organisms display strategies whereby substrate is “wasted” into byproducts, thereby lowering the biomass yield (high rate strategies). Indeed, *E. coli* [23] as well as the lactic acid bacterium *Lactobacillus plantarum* [96] achieve optimal *in silico* predicted growth when adapted on the poor substrate glycerol by serial dilution. However, on glucose, FBA predicted for *L. plantarum*, biomass yields, which were too high and incompatible with the observed lactate production [98]; similar discrepancies between model and experiment were observed for *E. coli* on glucose [23]. 

Due to the still growing number of genome-scale models across all kingdoms, FBA is a powerful tool to explore systemic properties of genome-scale metabolic networks for applications in biotechnology and medicine [5,99]. Nevertheless, caution should be taken with (i) the selection of objective function and (ii) non-uniqueness of the flux distributions that lead to optimisation of the objective function (*i.e.*,a whole solution space of flux distributions that is consistent with the prediction of the objective function). In addition, point (ii) can be addressed by, for instance, flux variability analysis (FVA) [100]. FVA is used to find the minimum and maximum flux values for the reactions in the network while maintaining some state of the network (e.g., supporting 90% of the objective function). 

### 4.2. Optimising the Predictive Power of FBA

The above examples point to an important aspect that obviously applies not only to FBA alone but also to all analyses that assume some kind of optimality criteria: the predictive power critically depends on the correspondence between the chosen objective function with the real biological objective. Indeed, it was shown for *E. coli* that, within the FBA formalism, different objective functions were needed to predict optimal flux states for different conditions [101]. The remaining part of this section will be used to discuss different extensions and constraints of FBA that are proposed to unravel the principles underlying the MRN. 

The surmise that protein expression and maintenance is a costly process has also penetrated the field of FBA. One way of implementing this is the minimisation of overall fluxes as the objective function. The rationale for this approach is the assumption that the magnitude of fluxes is related to the amount of protein required as catalyst of that flux. When applied to metabolic schemes of erythrocytes and *Methylobacterium extorquens* the fluxes predicted by the method were in good agreement with those calculated on the basis of a kinetic model [102,103]. Unfortunately, this objective too cannot explain why microorganisms switch between metabolic strategies due to the fact that the flux-minimisation algorithm will force some of the fluxes to zero if alternative “cheaper” reactions or pathways exist in the network. 

The optimality assumption underlying FBA may be valid for wild type organisms that have been evolved over many thousands of generations. It is less likely that this will also hold for engineered or mutant-strains. To overcome this, extensions of FBA that use different objective functions are proposed, such as ROOM [104] and MoMa [105]. Regulatory On/Off Minimisation (ROOM) aims to minimise the number of significant flux changes with respect to the wild type. This method is based on the assumption that upon a gene-knockout, the cost associated with adapting is minimised. In this manner, ROOM can be used to predict the metabolic state of an organism after a gene-knockout. Another variant of FBA called MoMA, which refers to the Minimisation of Metabolic Adjustment, seeks an approximate solution for a sub-optimal growth flux state, which is nearest in flux distribution to the unperturbed wild type. Both approaches have been shown to outperform FBA for certain specific examples [104,105]. Note, however, that both methods require a reference flux distribution from the wild type to predict fluxes in the mutant. 

In order to further improve the predictive power of FBA, there are options that do not act on the objective function, but on the constraints that limit the ways in which the objective can be reached. An example of an additional constraint is a molecular-crowding constraint. The rationale for this constraint comes from the limited amount of space inside cells available for metabolic enzymes [106]. As argued in Section 3.3, FBA fails to predict a metabolic switch (changing from high-yield to low-yield metabolic pathways with increasing substrate concentration). Recently, FBA extended with a molecular crowding constraint was used in a comparative study between three organisms (*E. coli*, *S. cerevisiae* and *L. lactis*) which differ in their metabolic strategies. With this extension, FBA was able to predict experimental findings and it was reported that different metabolic strategies could be attributed to the design principle of maintaining a redox balance within these organisms [107]. 

In the FBA formalism, constraints always act on the potential fluxes through reactions. First, one tries to include additional data that can be used to constrain the fluxes, particularly for cases where regulatory effects have a dominant influence on the behaviour of the organism’s metabolic fluxes. Covert *et al.* proposed an extension to FBA using regulatory information (regulatory FBA, rFBA) [108]. This was incorporated by means of a Boolean formalism, in which the state of a gene was represented as either transcribed or not transcribed in response to regulatory signals. Based on whether or not a gene was transcribed, the reactions catalysed by the corresponding enzymes were included or removed from the stoichiometric model. Although a good start to include regulatory interactions, the regulatory network was incomplete and imprecise. A better prediction can therefore be made when gene expression data is used to infer regulatory networks [109]. This approach was also successful to indicate knowledge gaps and identify previously unknown components and interactions in the regulatory and metabolic networks [110]. 

There have been a number of other approaches to augment regular FBA with regulatory constraints. Shlomi *et al.* [111] proposed steady-state regulatory FBA (SR-FBA). In addition to metabolic constraints, in SR-FBA there are four additional constraints defining the type of regulation. The combined functional state of the entire system in a given constant environment, referred to as metabolic-regulatory steady state (MRS), is described by a pair of consistent metabolic and regulatory steady states, which satisfy both the metabolic and regulatory constraints. Using the SR-FBA method, the authors were able to show that a considerable number of 36 genes are redundantly expressed, that is, they are expressed even though the fluxes of their associated reactions are zero. These 36 non-optimal fluxes could not have been identified with a classical FBA approach and reveals that cells maintain some sort of metabolic variability (or anticipatory behaviour) within a given growth medium. In another approach, Van Berlo *et al.* suggested applying changes in gene expression as soft constraints on changes in fluxes, *i.e.*, it is to be expected that if the expression of a gene is increased, its corresponding flux will also increase [112]. In their approach, flux distributions are sought that minimise the number of violations to this rule and it was shown that changes in gene expression are predictive for changes in fluxes. 

Other examples, originating from biophysical limitations, that are used as additional constraints are competition for membrane space [113] and grouping constraints based on genome context [114]. In the first study it is hypothesised that the outcome of a competition for membrane space between glucose transporters and respiratory chain proteins influences the ratio of respiration and fermentation. By incorporating a sole constraint based on this concept in the genome-scale metabolic model of *E. coli*, they were able to simulate respiro-fermentation [113]. The second study used three types of genomic context (conserved genomic neighbourhood, gene fusion events, and co-occurrence) to estimate the likelihood of an entire group of fluxes to be on or off. Predictions of a genome scale model were in good agreement with experimental data from *E. coli* [114]. 

FBA would greatly benefit from kinetic information of metabolic reactions. For some subnetworks, such as glycolysis, these kinetic models are available, and they have been integrated with FBA (iFBA) [115]. Focusing on the diauxic shift in *E. coli* the authors found a significant improvement over the individual rFBA and kinetic model. Such approaches have the potential to combine the best of two worlds: generating simulations which are more globally accurate and informative than the kinetic-based model, and more accurate in their details than the rFBA model alone. 

All approaches mentioned above, although coherent, still require further validation by datasets of steady-state flux distributions and microarray data, and so at this stage it is difficult to assess which of these methods is to be preferred. Finally, we would like to stress that predictions, true or false, are useful either way because these predictions are testable and therefore able to confirm or rule out hypotheses. 

## 5. Conclusion and Outlook

The overwhelming speed at which experimental data is currently generated has led to the recognition that the complexity of the underlying networks is enormous. In order to make sense of this data, systems biology is a scientific tool that can help in extracting design principles from it. In this review we have presented experimental and theoretical examples of this extraction procedure based on the premise that evolution has moulded biological networks to perform optimally. This assumption immediately raises the question: “optimised for what?”. This is an intriguing and difficult question to answer. Optimisation of an objective function is strongly related to cellular fitness (*i.e.*, the ability to generate offspring), but generally the link between fitness and molecular network properties is rather vague or indirect. However, for several networks, especially under the standard laboratory conditions, we have quite a good understanding of which underlying principles and mechanisms are responsible for optimising fitness; many examples have been discussed in this review. For other networks it remains to be shown whether the lack of understanding is due to (i) an incomplete topology of the network (e.g., missing regulatory interactions), (ii) a lack in experimental data, or (iii) finding the right objective functions and constraints. As argued throughout, we believe that focusing on points (i) and (iii) will shed most light on understanding biological behaviour. Furthermore, care should be taken that biological networks cannot be optimal at multiple objective functions at the same time. Multiple objectives have then to be traded-off, which can be studied by means of a Pareto front [87]. In our opinion, a better understanding of biological systems relies mainly on the identification and integration of objective functions and constraints that have shaped current biological networks. 
